# A bioreactor system for the manufacture of a genetically modified *Plasmodium falciparum* blood stage malaria cell bank for use in a clinical trial

**DOI:** 10.1186/s12936-018-2435-x

**Published:** 2018-08-06

**Authors:** Rebecca Pawliw, Rebecca Farrow, Silvana Sekuloski, Helen Jennings, Julie Healer, Thuan Phuong, Pri Sathe, Cielo Pasay, Krystal Evans, Alan F. Cowman, Louis Schofield, Nanhua Chen, James McCarthy, Katharine Trenholme

**Affiliations:** 1 Clinical Tropical Medicine Laboratory, QIMR Berghofer Medical Research Institute, 300 Herston Rd, Herston, Brisbane, QLD Australia; 2 School of Medicine, University of Queensland, Brisbane, Australia; 3 The Walter and Eliza Hall Institute of Medical Research, Melbourne, Australia; 4 Australian Institute of Tropical Health and Medicine, James Cook University, Cairns, Australia; 5 Department of Drug Resistance and Diagnostics, Australian Army Malaria Institute, Brisbane, Australia

**Keywords:** Malaria, *Plasmodium falciparum*, Bioreactor, In vitro cultivation, Good Manufacturing Practice

## Abstract

**Background:**

Although the use of induced blood stage malaria infection has proven to be a valuable tool for testing the efficacy of vaccines and drugs against *Plasmodium falciparum,* a limiting factor has been the availability of Good Manufacturing Practice (GMP)—compliant defined *P. falciparum* strains for in vivo use. The aim of this study was to develop a cost-effective method for the large-scale production of *P. falciparum* cell banks suitable for use in clinical trials.

**Methods:**

Genetically-attenuated parasites (GAP) were produced by targeted deletion of the gene encoding the knob associated histidine rich protein (*kahrp*) from *P. falciparum* strain 3D7. A GAP master cell bank (MCB) was manufactured by culturing parasites in an FDA approved single use, closed system sterile plastic bioreactor. All components used to manufacture the MCB were screened to comply with standards appropriate for in vivo use. The cryopreserved MCB was subjected to extensive testing to ensure GMP compliance for a phase 1 investigational product.

**Results:**

Two hundred vials of the GAP MCB were successfully manufactured. At harvest, the GAP MCB had a parasitaemia of 6.3%, with 96% of parasites at ring stage. Testing confirmed that all release criteria were met (sterility, absence of viral contaminants and endotoxins, parasite viability following cryopreservation, identity and anti-malarial drug sensitivity of parasites).

**Conclusion:**

Large-scale in vitro culture of *P. falciparum* parasites using a wave bioreactor can be achieved under GMP-compliant conditions. This provides a cost-effective methodology for the production of malaria parasites suitable for administration in clinical trials.

## Background

The use of controlled human malaria infection (CHMI) for testing the efficacy of vaccines and drugs against *Plasmodium falciparum* is now well established. Induced blood stage malaria (IBSM) infection was pioneered in the 1990s [1–3], and was subsequently refined [4–7]. A limiting factor in the IBSM system has been the restricted number of defined *P. falciparum* strains available. This is in part due to difficulties obtaining suitable material for IBSM studies. Until recently it has been necessary to rely on access to deliberately infected volunteers or malaria-infected travellers returning from overseas. In both cases, ethical approval is required to collect, store and use this material and there are concerns about contaminating infectious agents.

A process for culturing sufficient volumes of *P. falciparum* in tissue culture flasks for in vitro production of Good Manufacturing Practice (GMP) grade blood stage malaria cell banks was recently described [8]. This provides a culture-based alternative to sourcing parasites from individuals who have either experimental or natural infection (travellers returning from malaria endemic areas). This allows more flexibility in that new banks can be created as required, and not only when a suitable donor becomes available. It also allows for tight control over all components used in the cell bank manufacture and ensures that cell banks are renewable resources.

However, bulk preparation of *P. falciparum* cultures is both time consuming and technically demanding; yields can be low and parasite quality can be variable when grown in static culture. Multiply-infected erythrocytes (containing two or more parasites) are also observed more frequently than in suspension cultures [9]. The presence of erythrocytes containing multiple parasites is undesirable for IBSM studies where the parasite dose should be carefully controlled.

Dalton et al. have developed a method for production of large scale suspension cultures of asexual and sexual blood stage *P. falciparum* using a wave bioreactor (Wave Bioreactor™ 20/50 EHT system) [9, 10]. Growth of *P. falciparum* in the wave bioreactor system is superior to static flask cultures because parasites retain synchrony over at least 3 cycles of invasion. Additionally, the development of multiply infected erythrocytes is reduced; this was shown to be both consistent and reproducible [9, 10]. Disposable plastic bioreactors also allow for careful control and monitoring of the cellular environment, and have been widely used for the culture of plant, animal and microbial cells, and for the production of proteins, antibodies and other biologics conforming to GMP standards [11].

Here, the manufacture and release of a cell bank that complies with the GMP requirements for a phase 1 investigational product is described. The cell bank was manufactured using a wave bioreactor and consists of erythrocytes infected with genetically modified *P. falciparum* parasites. This cost-effective methodology using appropriate GMP compliant conditions allows for the production of sufficient quantities of material for IBSM studies and whole blood-stage vaccine trials, with sufficient material available for quality control and ongoing stability testing. The manufacture of the master cell bank was conducted in accordance with the United States Food and Drug Administration (FDA) Guidance for Industry: CGMP for Phase 1 Investigational Drugs.

## Methods

### Production of *Plasmodium falciparum* 3D7-KAHRP KO GAP pre-seed bank

Genetically attenuated parasites (GAP) were produced by targeted deletion of the gene encoding the knob-associated histidine-rich protein (*kahrp*) from *P. falciparum* strain 3D7, in a similar manner as previously described for this and other genes [12, 13]. Briefly, deletion of *kahrp* was achieved by a positive–negative selection strategy [14] whereby the entire *kahrp* gene was replaced through double cross-over homologous recombination with a cassette encoding human dihydrofolate reductase (*dhfr*), a selectable marker that renders transfectants resistant to WR99210 (Jacobus Pharmaceuticals, NJ).

The loxP-DHFR-loxP-pCC1 plasmid was created by insertion of a loxP-hDHFR-loxP cassette into the pCC1 plasmid backbone [15], as previously described [16]. The KAHRP-KO plasmid was created by cloning *kahrp* 5′ and 3′ sequences into this plasmid. Plasmid DNA was transfected into *P. falciparum* 3D7 by electroporation. Following WR positive selection, parasites were grown without WR to deselect those containing the WR-containing plasmid, thereafter WR was reapplied to select those with integration of the *dhfr* cassette into the genome. These were then placed under negative selection with 5–fluorocytosine (5-FC; Sigma) to select for homologous recombination. 5-FC resistant parasites were genotyped by PCR.

KAHRP-KO parasites were cloned by limiting dilution in 96-well flat bottom plates and the resulting clonal line, 3D7-KAHRP-KO, was characterized by whole genome sequencing (Accession Number PRJEB12838).

The *P. falciparum* 3D7 parasites used for transfection with the *kahrp* knockout vector were created from the 3D7 parental strain described previously [17]. This strain was used for the original IBSM studies [1] and has been extensively used for subsequent studies.

### Production of 3D7-KAHRP KO GAP seed bank

The GAP seed bank was produced by thawing the contents of one vial of the GAP pre-seed bank following a standard protocol [18] and expanding the parasites in culture (RPMI 1640 media supplemented with 10% heat-treated pooled human serum and 4% human erythrocytes) using standard methods [19]. At each passage, fresh complete media and erythrocytes were added and the culture was expanded to achieve the required volume for cell banking. When the culture contained a minimum of 80% ring-stage parasites at 3–5% parasitaemia, the seed bank was cryopreserved using Glycerolyte 57 (Fenwal Inc.) in a 1:2 ratio, aliquoted into 1 mL cryovials, and stored in vapour phase liquid nitrogen. The final GAP seed bank was transferred to a Therapeutic Goods Administration (TGA) licensed GMP facility (Q-Gen; QIMR Berghofer Medical Research Institute [QIMRB]) for further expansion and production of the GAP master cell bank.

### Production of 3D7 KAHRP-KO GAP master cell bank

**Fig. 1:**
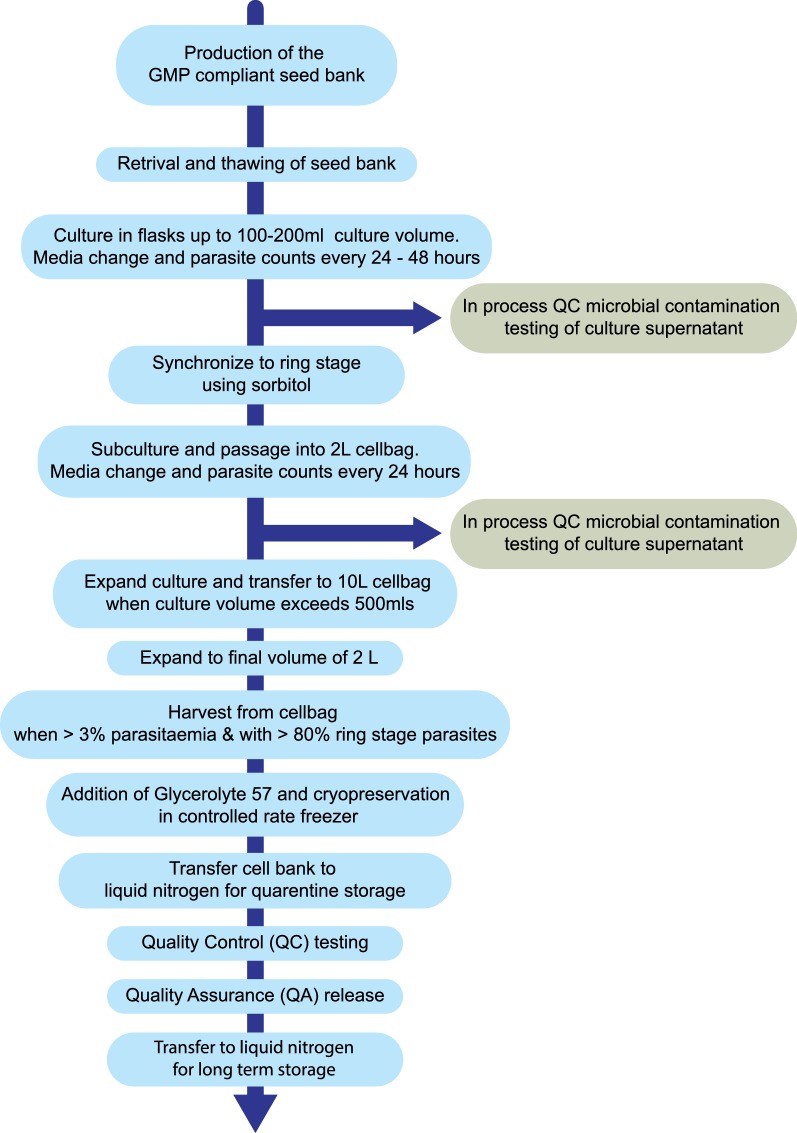
Process overview. The steps involved in production of the 3D7 KAHRP-KO GAP master cell bank

The 3D7 KAHRP-KO GAP master cell bank (GAP MCB) was manufactured in a ISO Class 5 Biological Safety Cabinet within an ISO Class 7 clean room using tissue culture flasks and a bioreactor (GE Wave 25™ System). The GAP MCB dossier was reviewed by the United States Food and Drug Administration (FDA) under the investigational new drug (IND) program. A summary of the process is shown in Fig. 1 and described in detail below.

### Parasite thaw and culture in flasks

A vial of the 3D7 KAHRP-KO GAP seed bank was thawed at 37 °C with the stepwise addition of 12 and 1.6% sodium chloride following a standard protocol [18]. The resulting red blood cell (RBC) pellet was washed in 0.9% sodium chloride and resuspended in 8 mL of complete RPMI-Hepes 1640 media (Life Technologies) containing 100 µL of 50% haematocrit (HCT) washed RBCs, and the cell suspension was transferred to a T25 cm^2^ flask.

Parasites were cultured in flasks under standard malaria culture conditions [19] in an atmosphere of 5% carbon dioxide, 5% oxygen and 90% nitrogen at 37 °C and media was changed every 24 h. Parasitaemia was monitored by examination of Giemsa-stained thin blood films. Additional media and fresh RBCs were added as required to maintain the culture at a parasitaemia of between 0.5 and 4%, and with a 2–5% HCT. The parasite culture was expanded to a volume of approximately 100 mL and the parasites were synchronized by a single treatment with 5% sorbitol [20] prior to transfer to a 2 L cellbag of the Wave 25™ Bioreactor.

### Parasite culture using wave bioreactor

**Fig. 2:**
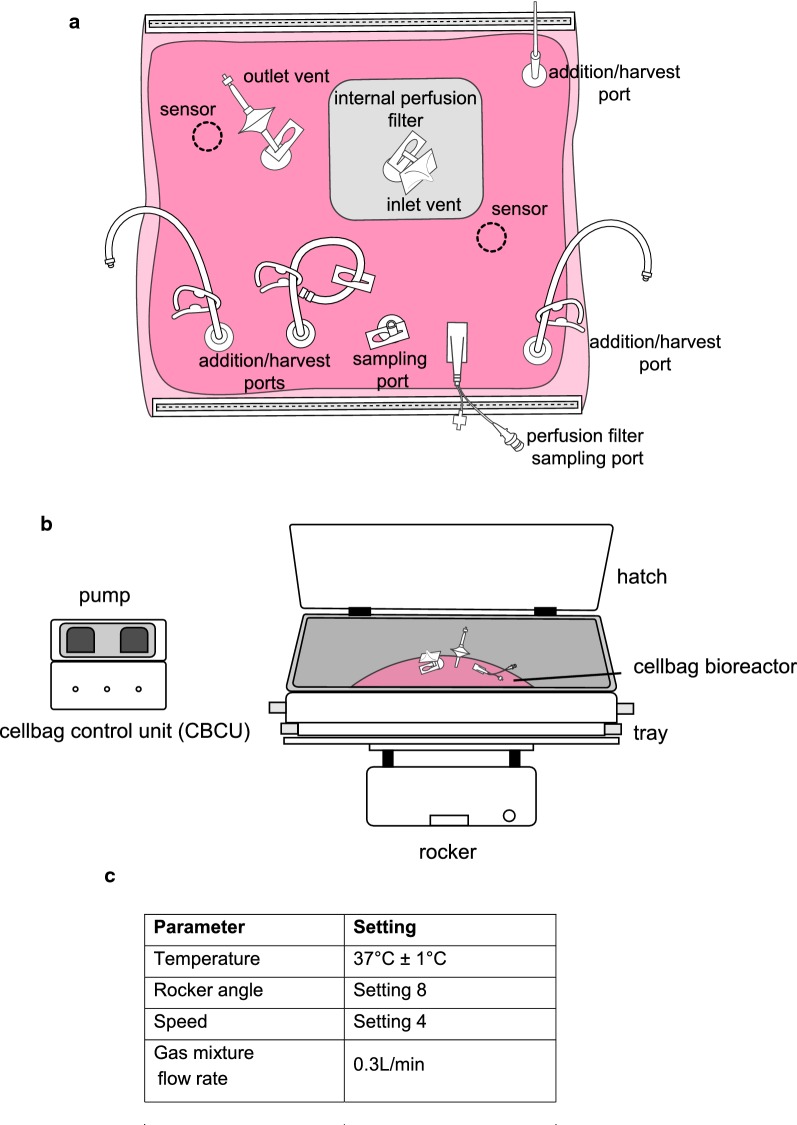
Wave bioreactor configuration. **a** Each cellbag has built-in inlet and outlet air filters, and ports that allow the addition of culture medium and extraction of samples. **b** The Ready to Process WAVE 25™ system (GE Healthcare) was used for this study. **c** Temperature, rocking speed, rocking angle and atmospheric conditions are monitored and controlled by the cellbag control unit (CBCU)

The Ready to Process WAVE 25™ system (GE Healthcare) was used for this study and configuration of the equipment is shown in Fig. 2. The system consists of a pre-sterile single use cultivation container (cellbag) made of FDA approved plastic. Each cellbag has built-in inlet and outlet air filters, and ports that allow the addition of culture medium and extraction of samples (Fig. 2a). The cellbag is placed on a temperature-controlled rocking platform (Fig. 2b); gas transfer and mixing of the cell culture is facilitated by movement of the rocker unit which induces a wave motion in the culture. Temperature, rocking speed, rocking angle and atmospheric conditions (Fig. 2c) are monitored and controlled by the cellbag control unit (CBCU). The system is controlled from a PC running UNICORN software version 6.3.2 or later.

The synchronous parasite culture was transferred into a 2 L cellbag and maintained under conditions controlled electronically by the bioreactor system (Fig. 2c). Parasitaemia was monitored by examination of Giemsa-stained thin blood films. The culture medium was changed daily, with washed RBCs added as required to maintain a parasitaemia of 0.5–1.2% and a 2–5% HCT. When the culture volume reached 500 mL it was transferred to a 10 L cellbag and the parasite culture was expanded to a final volume of approximately 2 L in line with the manufacturers recommended culture volumes for 2 and 10 L cellbags. To facilitate media change and transfer between cellbags the cell culture was removed from the in use cellbag under sterile conditions, centrifuged in 250 mL conical bottles (Corning) and the supernatant removed. The required volume of fresh media was pumped into the destination cell bag under the control of the CBC unit and the required volume of inoculum added under gravity flow.

The culture was monitored to ensure synchronicity and was maintained such that the % of ring stage parasites (as a % of the total parasites) present in the culture did not fall below 80%. When the required volume was reached, the culture was maintained for one further invasion cycle before parasites were harvested for banking at a parasitaemia target of > 3% and a minimum of 80% ring stage parasites. Progression through the final multiplication cycle was monitored by examination of Giemsa-stained thin blood films every 4–6 h. The cell culture supernatant was tested for microbial contamination prior to culture in the Wave 25™ Bioreactor system and prior to harvesting. At harvest the entire cell culture was removed under sterile conditions and centrifuged in 250 mL conical bottles to pellet the RBCs prior to freezing.

### Culture media

RPMI-Hepes 1640 media (Life Technologies) was supplemented with 10% heat inactivated (HI) pooled human serum and hypoxanthine (HT) supplement (0.1 mM sodium hypoxanthine and 0.016 mM thymidine). Complete media was used immediately or dispensed in aliquots and stored at a temperature of between 2 and 8 °C for a maximum of 7 days.

The serum used was supplied, screened and tested by Key Biologics (Memphis USA), an FDA registered blood collection and processing establishment. Whole blood units were collected from donors who were screened and found acceptable for donation of transfusable blood components based on all applicable FDA requirements. Infectious disease testing as required for qualification of blood components for transfusion to humans was carried out and was required to be non-reactive or negative. Serum from individual donors was tested (using FDA approved test kits) for human immunodeficiency virus (HIV)-I and HIV-II, human T-lymphotropic virus (HTLV) types I and II, hepatitis B virus (HBV), hepatitis C virus (HCV), *Trypanasoma cruzi* and syphilis (by serology), and underwent nucleic acid testing for HBV, HCV, HIV, and West Nile virus (WNV). Serum was then pooled from the multiple donors, frozen, stored and shipped under controlled conditions to QIMRB. On arrival the serum was thawed, heat inactivated at 56 °C, filtered and stored at − 20 °C in 50 mL aliquots until required. The pooled serum was additionally tested for Epstein Barr virus, Parvovirus B19 and herpes virus type 6 by PCR at Pathology Queensland. Endotoxin testing and sterility testing was performed at Q-Gen.

Leukodepleted packed RBCs (Group O Rh (D) negative) used for in vitro cultivation of *P. falciparum* parasites were obtained from the Australian Red Cross Blood Service (Blood Service). All supplied RBCs were screened according to Blood Service protocols to eliminate individuals with risk of exposure to transmissible spongiform encephalopathy and blood borne agents. The blood was collected and leukodepleted at the Blood Service and the donor’s blood sample was tested and certified non-reactive for HIV, HBV, HCV, HTLV and syphilis using diagnostic kits approved by the TGA. A blood sample was additionally tested by Pathology Queensland for infectious diseases that are not included in mandatory testing for blood and blood products including anti-Flavivirus IgM antibodies, *Trypanasoma cruzi* IgG antibodies, Barmah Forest virus IgM and IgG antibodies, and Ross River virus IgM and IgG antibodies. Samples were also screened (by PCR testing) for Epstein Barr virus (EBV), Cytomegalovirus (CMV), Parvovirus B19, Human Herpes virus type 6 and type 7(HHV-6 and HHV-7), and WNV. On receipt at QIMRB the cells were transferred from the Blood Service pack to a sterile container and a 2 mL aliquot was removed for sterility testing. Aliquots of RBCs were washed three times in RPMI and resuspended in complete RPMI to give a HCT of 50%. Blood was washed as required in aliquots not exceeding 20 mL and stored at 2–8 °C for up to 7 days after washing.

### Cryopreservation of the GAP MCB

Percentage parasitaemia and parasite life cycle stage were assessed by microscopic examination of thin blood films and when cultures achieved > 3% parasitaemia with at least 80% ring-stage parasites the cell bank was cryopreserved using Glycerolyte 57 in a 1:2 ratio (i.e. two volumes of Glycerolyte 57 were added to each volume of pRBC) as previously described [1].

The cell bank was dispensed in 1 mL aliquots in cryovials, cryopreserved using a controlled rate freezer as previously described [8] and stored in vapour phase liquid nitrogen at –196 °C. A total of 200 vials were produced; two vials were allocated as retention samples. The remainder of the cell bank vials were allocated for release testing. The release testing of the MCB complied with the requirements of the FDA’s Guidance for Industry- Characterization and Qualification of Cell Substrates and Other Biological Materials Used in the Production of Viral Vaccines for Infectious Disease Indications, the United States Pharmacopeia (USP) < 85 > for Bacterial Endotoxin Testing, USP < 63 > mycoplasma/spiroplasma testing, and the British Pharmacopoeia Appendix XVI A: Test for Sterility. The parameters assessed, acceptance criteria, test methods and place of testing are listed in Table 1.

**Table 1:** Specifications for the GAP MCB

Assay	Specifications (acceptance criteria)	Test methods	Performed by
Red cell pellet	> 50 μL of RBC pellet is required after thawing the 3D7 KAHRP-KO GAP MCB and centrifuging the RBC suspension	Measured using a calibrated pipette	QIMR-B
Identification	Absence of *kahrp* gene	*P. falciparum* 3D7 KAHRP-KO GAP Verification	QIMR-B
Percent parasitaemia	> 3% parasitized RBC ≥ 80% ring stage parasite	Malaria thin film smears	QIMR-B
Percent viable (active cell frequency) parasites	≥ 25% viable	Limiting dilution assay	QIMR-B
Sterility	No growth	Microbial Contamination and Sterility Testing (SOP QC-11) British Pharmacopoeia (BP) 2015, Appendix XVI A: test for sterility	Q-Gen
Endotoxin Testing	< 5 EU/mL	Lonza’s Limulus Amebocyte Lysate (LAL) PYROGENT™ Ultra Assay	Q-Gen
Mycoplasma	No mycoplasma detected	Test for the detection of mycoplasma including qualification of the test article in accordance with European pharmacopeia, section 2.6.7 mycoplasmas	BioReliance
Spiroplasma culture	Not detected	Test for the detection of agar cultivable Spiroplasma including qualification of test article in accordance with US Pharmacopeia < 63 > mycoplasma/spiroplasma testing	BioReliance
HIV 1 and 2	Not detected	Real time PCR assay for the detection of HIV I and HIV II in biological samples	BioReliance
HTLV I and II	Not detected	Pinnacle Q-PCR™ assay for the detection of HTLV	BioReliance
EBV	Not detected	Pinnacle Q-PCR™ assay for the detection of PCR for viral genome	BioReliance
CMV	Not detected	Pinnacle Q-PCR™ assay for the detection of PCR for viral genome	BioReliance
Hepatitis C Virus	Not detected	Pinnacle Q-PCR™ assay for the detection of PCR for viral genome	BioReliance
Hepatitis B Virus	Not detected	Pinnacle Q-PCR™ assay for the detection of PCR for viral genome	BioReliance
Human Parvovirus (B19)	Not detected	Pinnacle Q-PCR™ assay for the detection of PCR for viral genome	BioReliance
West Nile virus (WNV)	Not detected	Real time PCR for the detection of WNV in biological samples	BioReliance
Adeno-associated virus (AAV)	Not detected	Pinnacle Q-PCR™ assay for multiple serotypes of adeno-associated viruses (AAV) pathogen detection	BioReliance
Human Herpes virus type 7	Not detected	Pinnacle Q-PCR™ assay for the detection of PCR for viral genome	BioReliance
Human Herpes virus type 6	Not detected	Pinnacle Q-PCR™ assay for the detection of PCR for viral genome	BioReliance
Retroviruses	Report Result	Evaluation of reverse transcriptase activity by ultracentrifugation and quantitative fluorescent product enhanced reverse transcriptase (QPERT) assay	BioReliance
Quantitative transmission electron microscopy of sections for the detection of viruses, fungi, yeasts, bacteria and mycoplasmas (200 cell profiles)	No extraneous agents observed in the 200 cell profiles examined	Quantitative transmission electron microscopy of sections for detection of viruses, fungi, yeasts, bacteria and mycoplasmas (200 cell profiles)	BioReliance
Presence of viral contaminants	Not detected	In vitro assay for the detection of viral contaminants using 3 detector cell lines	BioReliance
Presence of inapparent viruses	Not detected	Test for the presence of inapparent viruses using suckling mice, adult mice, guinea pigs and embryonated eggs in accordance with FDA CBER guidance	BioReliance

### Parasite viability following cryopreservation

Parasite viability post-freezing was determined by measurement of cloning efficiency via a limiting dilution assay [8, 21] with positivity determined by production of *P. falciparum* histidine-rich protein II (HRP-II).

In order to determine post-freeze viability, a vial of the GAP MCB was thawed following a standard protocol [18] and a sample was removed for RBC counting using a haemocytometer. The number of parasitized/infected RBCs (pRBCs) was determined via microscopic examination of a Giemsa-stained thin blood smear.

Dilutions of pRBCs were prepared in complete media and dispensed in 100 μL aliquots in 96-well flat bottom plates at 2% HCT at theoretical concentrations of 40, 20, 10, 5, 2.5, 1.25, 0.625 and 0.3125 parasites/well. Thirty-two replicates of each concentration were plated out and non-parasitized RBCs were included as a negative control. Plates were incubated in an atmosphere of 5% O_2_, 5% CO_2_ and 90% N_2_ at 37 °C for 7 days and media was replenished on Day 4. On Day 7, supernatant was removed from each well and processed immediately or frozen for future testing. The supernatant was tested for *P. falciparum* HRPII using the SD Malaria Antigen Pf ELISA kit, according to the manufacturer’s instructions.

Briefly, supernatants from each well were transferred to the corresponding well of an uncoated microplate and incubated for 15 min following the addition of 150 µL enzyme conjugate (anti-mouse *P. falciparum* HRP II Ig conjugated to horseradish peroxidase in lysis buffer). A 100 µL sample was then transferred from each well of the uncoated microplate to the corresponding well of the pre-coated assay microplate and incubated. Plates were washed and 100 µL of tetramethylbenzidine substrate added to each well. After a 10 min incubation, the reaction was stopped by the addition of 100 µL of 1 mL/L (1 N) hydrochloric acid. The absorbance was read at 450 nm with reference wavelength at 620 nm. The mean absorbance values (optical density [OD]) for the positive and negative controls were calculated in order to ensure the validity of the assay.

The cut off value for parasite positivity was calculated using the absorbance values of the 32 two wells containing the RBC negative control. The cut off value was the mean plus 3 standard deviations and this value was applied to the test samples to calculate the number of positive and negative wells. Limiting dilution analysis was performed to determine the frequency of viable parasites as previously described [22].

### In vitro anti-malarial drug sensitivity testing of the GAP MCB

Anti-malarial drug sensitivity testing was carried out on the GAP MCB parasites using the standard [^3^H]-hypoxanthine uptake inhibition assay [23] to ensure that the drug resistance phenotype of the parasites had not changed during culture. The in vitro sensitivity of the GAP MCB parasites to nine anti-malarial drugs was assessed (chloroquine, dihydroartemisinin, piperaquine, lumefantrine, amodiaquine, atovaquone, pyronaridine, mefloquine, quinine). The parental 3D7 line (chloroquine sensitive) and W2 (chloroquine resistant) were included as controls. The assays were performed at the Army Malaria Institute, Queensland, Australia.

Briefly, aliquots of parasites from the GAP MCB were grown in vitro and synchronized to give 95% ring stage parasites. The parasite culture was dispensed into a 96-well plate containing twofold dilutions of each anti-malarial drug for testing. [^3^H]-hypoxanthine was added and the amount incorporated into parasites was measured after a 48-h incubation. The IC_50_ values for each drug were determined by nonlinear regression analysis. This calculation was performed using GraphPad Prism software (GraphPad Prism version 6.00). Drug threshold values were expressed as mean IC_50_ (95% confidence interval) in nmol/L. The results were compared to published threshold values for drug resistance [24].

### Genotyping of the GAP MCB

The genotype of the GAP MCB was determined by PCR using primers to confirm KAHRP gene deletion and hDHFR integration. In vitro culture samples were taken at the beginning of the process (Day 0) and at harvest (Day 16) and placed in Qiagen lysis buffer. Genomic DNA was extracted from paired samples using a Qiagen DNAmp DNA Blood Mini Kit and gDNA from the parent strain 3D7 was used as a control. Standard PCR was set up using 4 pairs of primers (Table 2) in PCR mix as follows:

**Table 2:** PCR primers used for this study

Oligo name	Sequence	
Primers used for Genotyping of the GAP MCB		
mo517	GGAACTCATTAATATGTATG	
mo518	CAAAACCCATACTAAAAAAG	
mo519	GAGAACTTTAGCACAAAAGC	
mo520	TTTACGCTTTCTGCATCTTC	
Aw171	CCTAATCATGTAAATCTTAAATTTTTC	
Aw560	CCAATAGAT AAAATTTGTAG	

Oligo name	Sequence	Target
Primers used for qRT PCR		
KAHRP-FB	CATATAGTGCAATAATGGAAACGGA	*Pf* KAHRP gene (inside excised region)
KAHRP-RB	GGTGATTTACTTCTCCATGATGATG	
KAHRP-F	TGTTCCAGCAGATGCACCAA	*Pf* KAHRP gene (in remaining region)
KAHRP-R	GAGCTGAATAGCCTGCACCA	
MDR1-T1 F	TATGCATTTGTGGGAGAATCAG	*Pf* MDR1 [*multi drug resistance*] (single copy gene reference)
MDR1-T1R	CTCCTTCGGTTGGATCATAAAG	
hDHFR (FG)	ATGCATGGTTCGCTAAACT	hDHFR [*human dihydrofolate reductase*] gene
hDHFR R1	CCAGGTCTTCTTACCCATAAT	
hDHFR FA	CCTAATAGAAATATATCAGGATCCAT	hDHFR (recombination region)
hDHFR1 R	GGTCTTCTTACCCATAATCA	
LDH-T1F	AGGACAATATGGACACTCCGAT	*Pf* LDH1[*lactate dehydrogenase*] (single copy gene reference)
LDH-T1R	TTTCAGCTATGGCTTCATCAAA	
Pf SARS F1	AAGTAGCAGGTCATCGTG	*Pf* SARS [*seryl*-*tRNA synthetase*] (housekeeping gene)
Pf SARS R1	CGGCACATTCTTCCATA

**Mix 1.**: Primers **mo517** and **mo518**. No band expected when used with gDNA from *P. falciparum* 3D7 KAHRP-KO.
**Mix 2.**: Primers **mo517** and **Aw560** Presence of a 1463 bp band expected when used with gDNA from *P. falciparum* 3D7 KAHRP-KO.
**Mix 3.**: Primers **mo519** and **mo520** No band expected when used with gDNA from *P. falciparum* 3D7 KAHRP-KO.
**Mix 4.**: Primers **Aw171** and **mo520** Presence of 1128 bp expected when used with gDNA from *P. falciparum* 3D7 KAHRP-KO.

Amplification of the PCR product was performed using *Taq* DNA polymerase under the following cycling conditions: One initial cycle at 45 °C for 15 min, denaturing at 94 °C for 30 s, annealing at 52 °C for 40 s, extension at 68 °C for 105 s (30 cycles), one final extension cycle at 68 °C for 7 min.

### Quality check of GAP MCB by qRT-PCR

**Fig. 3:**
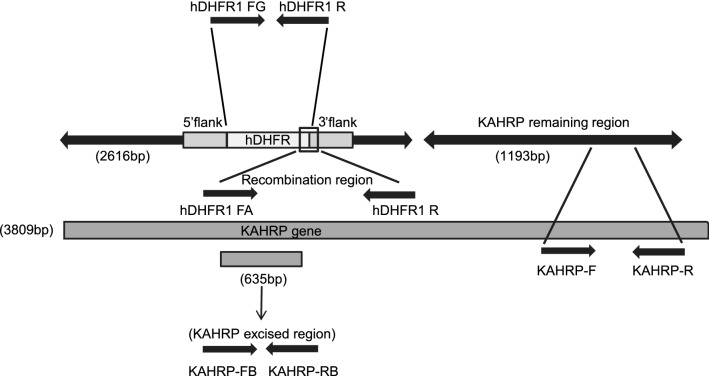
qRT-PCR primers mapped on KAHRP gene. To validate the successful genetic attenuation of *P. falciparum* 3D7 and verify hDHFR integration, a real time PCR assay was set up using primers designed at various regions of the *P. falciparum* 3D7 GAP

To further validate the successful genetic attenuation of *P. falciparum* 3D7 and verify hDHFR integration, a real time PCR assay was set up using primers designed at various regions of the *P. falciparum* 3D7 GAP (Fig. 3). Primer sequences are listed in Table 2.

To determine limit of detection of possible contamination with wildtype *P. falciparum* 3D7, two single copy genes (*pfmdr*-1 and *pfldh*) were amplified and used as reference for copy number estimation. A standard curve containing five ten-fold serial dilutions of wildtype *P. falciparum* 3D7 was prepared, starting from 2.0 ng/μL to amplify KAHRP excised region and MDR1 and LDH genes.

Triplicate PCR reactions were set up using the Roche Fast Start Essential DNA Green Master Mix (Cat. No. 06 402 712 001), 10 μM of each primer (Table 2) and 1 μL of each gDNA sample in a 12 μL reaction volume. Quantitative PCR was performed using a Roche Light Cycler 96 with the following cycling conditions: 94 °C for 3 min; 45 cycles of 94 °C for 30 s, 55 °C for 30 s and 68 °C for 30 s, and one final extension cycle of 68 °C for 5 min.

### Other testing performed on the Gap MCB

A summary of testing performed on the GAP MCB is shown in Table 1. Sterility testing complying with the requirements of the British Pharmacopoeia (BP) Appendix XVI A: Test for Sterility was carried out using direct inoculation as well as testing the supernatant following membrane filtration. Endotoxin inhibition testing of the GAP MCB and Glycerolyte 57 was performed to comply with the requirements of USP < 85 > Bacterial Endotoxin Test. Testing for Adventitious Agents complied with the requirements outlined in the FDA’s Guidance for Industry: Characterization and Qualification of Cell Substrates and Other Biological Materials Used in the Production of Viral Vaccines for Infectious Disease Indications. Assays for HIV 1 and 2, HTLV I&II, EBV, CMV, HCV, HBV, human parvovirus (B19), West Nile virus (WNV), adeno-associated virus (AAV), human herpes virus type 7, human herpes virus type 6 and retroviruses were performed in addition to testing for spiroplasma and mycoplasma. Sections were examined by quantitative transmission electron microscopy for the detection of viruses, fungi, yeasts, and bacteria (200 cell profiles). In vitro assays for the detection of viral contaminants and inapparent viruses was also performed.

## Results

A cell bank consisting of erythrocytes infected with genetically-modified *P. falciparum* parasites was successfully manufactured using a wave bioreactor. Two hundred vials of the GAP MCB were produced that met the requirements for use in Phase I clinical trials.

### Growth of *P. falciparum* 3D7-KAHRP KO GAP in a bioreactor

**Fig. 4:**
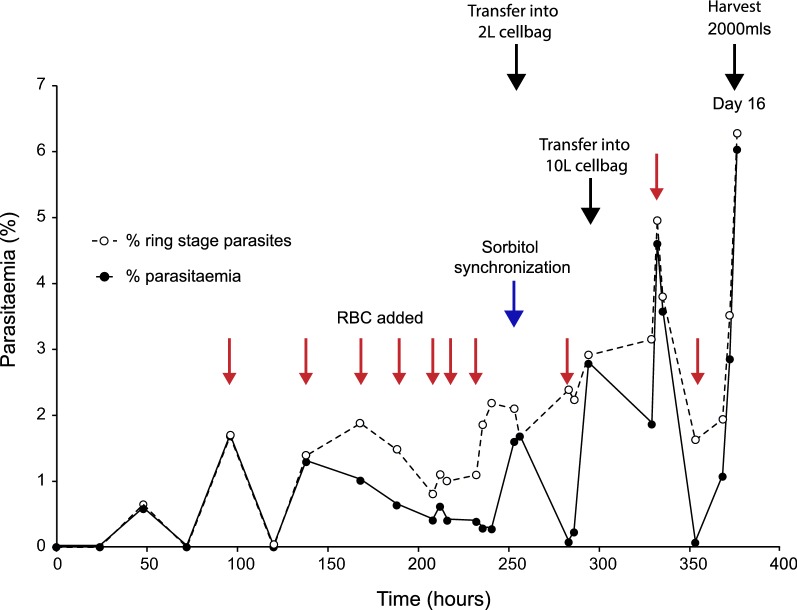
Profile of parasitaemia and parasite life cycle stages during growth in biowave reactor system. Parasites maintained an asexual blood stage cycle of approximately 39 h during the in vitro culture period, and showed a normal maturation pattern. The percent parasitaemia and percent ring stage parasites of the culture over time were determined by microscopy

**Fig. 5:**
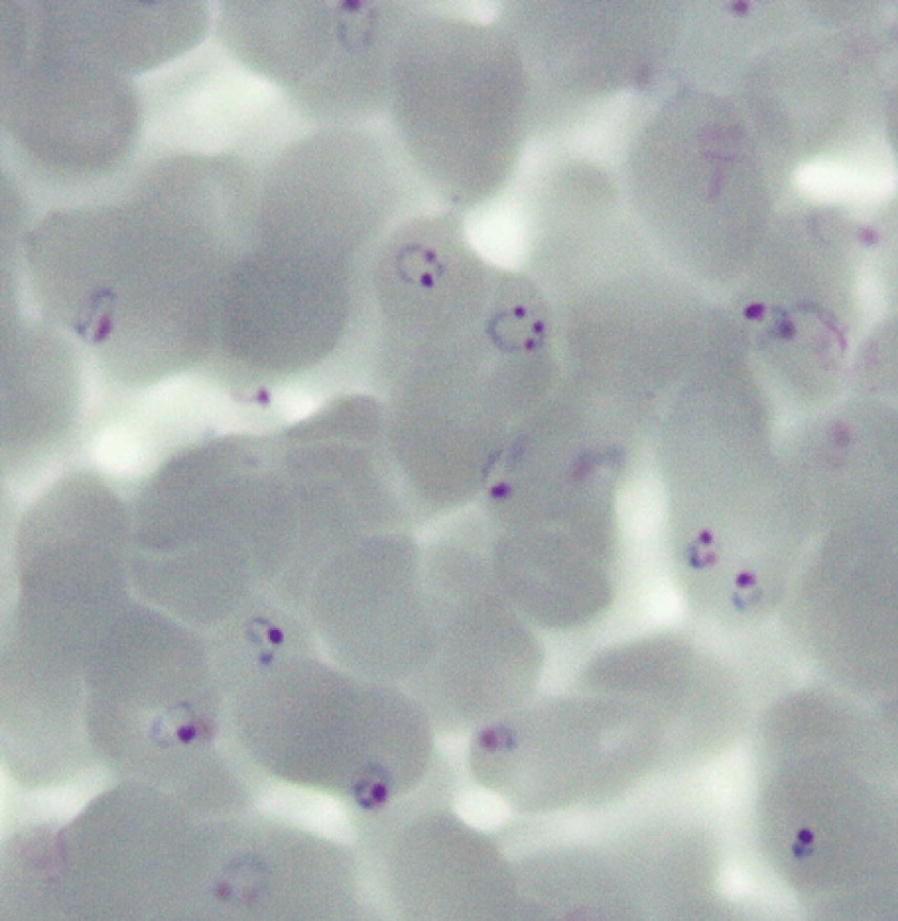
Giemsa-stained smear showing GAP MCB parasites at time of cryopreservation. At harvest the culture contained a high percentage of ring stage parasites

GAP parasites maintained an asexual blood stage cycle of approximately 39 h during the in vitro culture period in the bioreactor, and showed a normal maturation pattern. The percent parasitaemia and percent ring stage parasites of the culture over time is illustrated in Fig. 4. Parasitaemia was maintained between 0.5 and 2% by the addition of RBCs during the expansion phase (Day 0–10). During the banking phase (Day 10–15) the parasitaemia was maintained below 4%. At harvest the culture had a parasitaemia of 6.3%, with 96% of parasites at ring stage (high percentage of ring stage parasites is illustrated in Fig. 5). Thus, the pre-defined acceptable criteria of > 3% parasitized RBC and ≥ 80% ring stage parasites (Table 1) were met.

### Identity testing of the GAP MCB

**Fig. 6:**
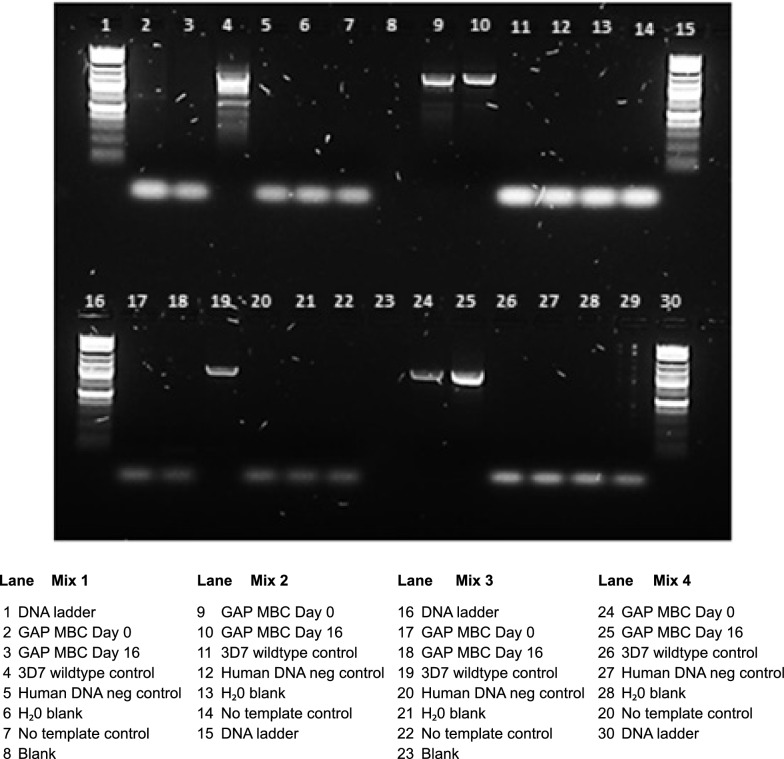
Agarose gel showing the genetic identity of the GAP MCB at Days 0 and 16. PCR assays confirmed *kahrp* gene deletion and hDHFR integration in parasite samples collected at the initiation of culture (Day 0) and at harvest (Day 16)

PCR assays confirmed *kahrp* gene deletion and hDHFR integration in parasite samples collected at the initiation of culture (Day 0) and at harvest (Day 16) (Fig. 6) demonstrating that the genotype of the parasites was maintained during the culture and banking process.

### Quality check of *Pf* KAHRP-KO by qRT-PCR

Mean Ct values using primer pairs for KAHRP (inside remaining region), hDHFR and recombination region were < 31 (positive) using KAHRP-KO gDNA as template indicating success of integration. Mean Ct values were > 31 (negative) using *P. falciparum* 3D7 (wildtype) gDNA as template. Mean Ct value using primer pairs for KAHRP gene (in excised region) was < 31 (positive) only for *P. falciparum* 3D7 (wildtype) gDNA. Mean Ct values were > 31 (negative) or undetectable (no Ct) for KAHRP-KOa and KAHRP-KOb gDNA as templates indicating success of deletion in the genetically attenuated parasite.

Amplification of the KAHRP excised region on serially diluted wildtype *P. falciparum* 3D7 and comparing Ct values obtained from serially diluted GAP showed undetectable contamination of the GAP starting from 2.0 ng/μL DNA concentration up to as low as 2.0 × 10^−5^ ng/μL. This is equivalent to a single copy or less using single copy gene references *pfmdr*-1 and *pfldh*.

### In vitro drug sensitivity of GAP MCB parasites

The in vitro sensitivity of the GAP MCB parasites to a panel of 9 anti-malarial drugs is shown in Table 3. The mean IC_50_ (95% confidence interval) results for each drug are presented in Table 4 along with the corresponding results for the control P*. falciparum* strains 3D7 and W2.

**Table 3:** In-vitro drug sensitivity profile of the GAP MCB parasites

Drug	Control		Sample
	3D7	W2	GAP MCB
Chloroquine	S	R	S
Dihydroartemisinin^a^	S	S	S
Piperaquine	S	S	S
Lumefantrine	S	S	S
Amodiaquine	S	S	S
Atovaquone^a^	S	S	S
Pyronaridine^a^	S	S	S
Mefloquine	R	S	R
Quinine	S	R	S

*S* drug sensitive, *R* drug resistant

^a^Insufficient in vivo data to validate in vitro results; no established threshold values

**Table 4:** Drug threshold values for GAP MCB parasites

Anti-malarial drug	Test 1			Test 2			Test 3		
	Control		Test sample	Control		Test sample	Control		Test sample
	3D7	W2	GAP MCB	3D7	W2	GAP MCB	3D7	W2	GAP MCB
*CQ (chloroquine)*									
IC50 (nM)	14.85	206.8	14.37	16.53	297.6	13.77			
95% confidence intervals (nM)	(8.489–25.98)	(131.6–325.0)	(8.671–23.80)	(9.880–27.64)	(179.3–493.9)	(8.149–23.28)			
*DHA (dihydroartemisinin)*									
IC50 (nM)	5.624	4.354	4.931	7.366	2.019	7.943			
95% confidence intervals (nM)	(4.271–7.406)	(2.902–6.532)	(3.667–6.629)	(5.600–9.689)	(1.539–2.649)	(6.065–10.40)			
*PQ (piperaquine)*									
IC50 (nM)	18.15	46.02	15.27	30.69	48.85	23.59			
95% confidence intervals (nM)	(10.41–31.66)	(29.78–71.11)	(8.490–27.47)	(19.33–48.72)	(34.94–68.31)	(15.29–36.39)			
*LF (lumefantrine)*									
IC50 (nM)	No results	70.35	No results	No results	42.32	No results	315.2	25.31	335.8
95% confidence intervals (nM)		(46.35–106.8)			(33.26–53.85)		(205.2–484.3)	(18.11–35.38)	(251.3–448.7)
*AQM (desethylamodiaquine)*									
IC50 (nM)	10.19	66.83	10.35	18.46	83.88	15.55			
95% confidence intervals (nM)	(6.031–17.21)	(40.88–109.3)	(5.551–19.31)	(10.06–33.86)	(48.10–146.3)	(9.139–26.47)			
*ATQ (atovaquone)*									
IC50 (nM)	0.9587	1.939	1.147	0.7122	1.524	1.216			
95% confidence intervals (nM)	(0.8493–1.082)	(1.752–2.147)	(0.9806–1.342)	(0.6224–0.8150)	(1.304–1.781)	(1.094–1.352)			
*PD (pyronaridine)*									
IC50 (nM)	5.398	9.897	7.804	27.67	21.88	12.59			
95% confidence intervals (nM)	(3.359–8.675)	(5.615–17.45)	(3.473–17.54)	(15.88–48.19)	(11.82–40.49)	(6.720–23.57)			
*MQ (mefloquine HCl)*									
IC50 (nM)	101.5	15.32	98.87	157	12.26	61.27			
95% confidence intervals (nM)	(72.61–141.8)	(10.85–21.65)	(69.17–141.3)	(79.04–311.8)	(8.573–17.53)	(43.67–85.96)			
*QN (quinine HCl)*									
IC50 (nM)	203.2	795.6	227.4	335.6	569.6	89.73			
95% confidence intervals (nM)	(169.9–243.0)	(527.6–1200)	(173.1–298.8)	(195.2–577.1)	(432.1–750.9)	(62.87–128.1)			

Drug threshold values are expressed as mean IC50 (95% confidence interval) in nmol/L

The assay was repeated twice and reproducible results were obtained

A third assay was performed for lumefantrine as IC50 values were not detectable in the first two assays

GAP master cell bank parasites demonstrated sensitivity to eight of the anti-malarial drugs tested (chloroquine, artemisinin, piperaquine, lumefantrine, amodiaquine, atovaquone, pyronaridine, quinine) and resistance to mefloquine.

### Parasite viability of the GAP MCB following cryopreservation

Parasite viability was tested using samples taken approximately 24 h (Time 0) after freezing and at 3 months post-production. The GAP MCB was determined to contain 52% viable parasites at Time 0 and 64% viable parasites at 3 months post-production. These values conform to the pre-defined acceptable criteria of > 25% viable parasites. The variability between the two time points is within normally observed limits for this assay.

## Discussion

Our aim was to produce a GMP compliant cell bank consisting of KAHRP deficient *P. falciparum* parasites using the wave bioreactor system. Previous studies have shown that the wave bioreactor system is suitable for the production of large volumes of high quality *P. falciparum* cultures [9]. We have taken this method further to demonstrate that the wave bioreactor can be used to culture parasites for use in phase 1 clinical trials.

The use of a wave bioreactor system has a number of significant advantages over traditional culture of *P. falciparum* in flasks. Foremost is that it allows close control of culture conditions that can be monitored and maintained electronically by the wave bioreactor system; the cellbags used are single use closed systems approved by the FDA. Significant savings in time and labour were also achieved; 100 mL of culture was transferred to a cellbag on Day 11, expanded to 500 mL on Day 12, and a culture volume of 2 L was achieved on Day 15 with harvest/cryopreservation on Day 16. During this rapid expansion phase the labour required to maintain the culture (monitor parasitaemia, change media, add fresh RBCs) was less than would be required if growing in multiple tissue culture flasks. In addition, the use of multiple flasks can result in cultures spending more time in less than optimal growth conditions, and increase the probability of contamination.

The continual wave motion of the wave bioreactor ensures gentle movement of cells in culture resulting in a high proportion of single infections [9]. This allows for accurate calculation of parasite numbers which is essential for defined dose delivery in clinical trials. Results confirmed that wave bioreactor cultures contained high quality ring stage parasites with a low number of multiply infected red cells (< 0.5%). Additionally, the effective cryopreservation of the MCB was demonstrated by the fact that parasite viability was over 50% after 3 months.

The ability to use research grade starting material, including genetically-modified parasites, to produce a GMP grade product offers significant opportunities. It allows for genetically-modified parasites to be used as vaccine candidates and for the number of *P. falciparum* strains available for IBSM studies to be expanded. The availability of multiple antigenically and geographically diverse *P. falciparum* strains for IBSM studies is essential to allow testing of new anti-malarials against multiple strains before field testing begins.

The methodology described here is highly cost effective, with each of the 200 vials produced containing sufficient material for 3600 doses at an approximate cost of USD 0.14/dose. Although the method requires use of a specialist item of equipment (wave bioreactor system), the fact that these units are becoming widely used for a range of applications (including tissue engineering, cellular and gene therapies, the production of therapeutic proteins and human vaccines) means that they are frequently available within research facilities or hospitals. The use of sterile, single use closed system cell bags means that a wave bioreactor system can be shared between groups without risk of cross contamination.

This methodology may represent a cost effective strategy by allowing extensive testing in a controlled environment (phase 1 trials) before deciding to move to field studies (phase 2 trials) which can be very expensive due to logistic and administrative challenges associated with carrying out clinical trials in a distant research site. The limiting factor for this methodology is that it is currently only useful for *P. falciparum* as other human malaria species are not easily grown in vitro.

## Conclusion

The large scale in vitro culture of *P. falciparum* parasites using a wave bioreactor can be achieved under GMP compliant conditions. This provides a cost-effective methodology for the production of malaria parasites suitable for administration in clinical trials.

## Abbreviations

IBSM: induced blood stage malaria
GMP: Good Manufacturing Practice
GAP: genetically-attenuated parasites
kahrp: knob-associated histidine-rich protein
MCB: master cell bank
FDA: Food and Drug Administration
CHMI: controlled human malaria infection
dhfr: human dihydrofolate reductase
5-FC: 5-fluorocytosine
TGA: Therapeutic Goods Administration
QIMRB: QIMR Berghofer Medical Research Institute
GAP MCB: 3D7 KAHRP-KO GAP master cell bank
IND: investigational new drug
RBC: red blood cell
HCT: haematocrit
HI: heat inactivated
HT: hypoxanthine
HIV: human immunodeficiency virus
HTLV: human T-lymphotropic virus
HBV: hepatitis B virus
HCV: hepatitis C virus
WNV: West Nile virus
Blood Service: Australian Red Cross Blood Service
EBV: Epstein Barr virus
CMV: Cytomegalovirus
OD: optical density
pRBC: parasitized/infected RBCs
HRP-II: histidine-rich protein II
